# Dendritic cell therapy for neurospoagioma: Immunomodulation mediated by tumor vaccine

**DOI:** 10.1038/s41420-023-01782-7

**Published:** 2024-01-06

**Authors:** Da Qian, Yuxiang Liu, Jie Zheng, Jinquan Cai

**Affiliations:** 1 Department of Burn and Plastic Surgery-Hand Surgery, Changshu Hospital Affiliated to Soochow University, Changshu No.1 People’s Hospital, Changshu, 215500 Jiangsu Province China; 2 Department of Neurosurgery, The Second Affiliated Hospital of Harbin Medical University, 150086 Harbin, China

**Keywords:** CNS cancer, Diseases

## Abstract

Neurospagioma, arising from different glial cells such as astrocytes, oligodendrocytes, and ependymal cells, stands as the prevalent intracranial tumor within the central nervous system. Among its variants, glioblastoma (GBM) represents the most aggressive form, characterized by a notably high occurrence rate and a discouragingly low survival prognosis. The formidable challenge posed by glioblastoma underscores its critical importance as a life-threatening ailment. Currently, clinical approaches often involve surgical excision along with a combination of radiotherapy and chemotherapy. However, these treatments frequently result in a notable recurrence rate, accompanied by substantial adverse effects that significantly compromise the overall prognosis. Hence, there is a crucial need to investigate novel and dependable treatment strategies. Dendritic cells (DCs), being specialized antigen-presenting cells (APCs), hold a significant position in both innate and adaptive immune responses. Presently, DC vaccines have gained widespread application in the treatment of various tumors, including neurospoagioma. In this review, we summarize the immunomodulatory effects and related mechanisms of DC vaccines in neurospoagioma as well as the progress of clinical trials to propose possible challenges of DC vaccines and new development directions.

## Fact

- DC vaccines used for targeted anti-tumor purposes can be broadly categorized into two types: DC polypeptide vaccines and DC gene vaccines.
- DC vaccines are involved in regulating the immune response in neurospoagioma.
- DC vaccines have demonstrated their potential in modulating the immune response against neurospoagioma.

## Open question

- What are the mechanisms by which DC vaccine regulates the immune response of neurospoagioma?
- How can vaccine formulation be optimized to improve immunotherapy with neurospoagioma?
- Whether the DC vaccine can be combined with other drugs to treat neurospoagioma?

## Introduction

Neurospoagioma, which originates from various glial cell types including astrocytes, oligodendrocytes, and ependymal cells, is the most prevalent type of central nervous system tumor. This tumor is marked by rapid proliferation, limited differentiation, significant invasiveness, postoperative recurrence, and an unfavorable prognosis [1]. Glioblastoma (GBM), comprising 56.6% of all neurospoagiomas, represents the most aggressive form of this tumor. Characterized by a high incidence and a low survival rate, GBM poses a significant life-threatening condition [2]. Currently, clinical practice frequently employs surgical excision along with combined radiotherapy and chemotherapy. However, this approach is plagued by a high recurrence rate, severe adverse reactions, and a substantial negative impact on the overall prognosis [3]. Hence, it becomes imperative to delve into novel and dependable treatment avenues. In recent times, among the myriad emerging therapies, active immunotherapy has emerged as the most promising and captivating focal point in contemporary oncology interventions. Tumor immunotherapy holds a central position as a hotspot and developmental trajectory in the realm of treating malignant neurospoagioma [4].

Tumor immunotherapy essentially involves a dynamic interplay between the immune system and the tumor. As early as 2004, Dunn et al. [5] Systematically summarized this process and proposed the “3Es hypothesis”, that is, immune cells and tumor cells constantly struggle in “Elimination”, “Equilibrium” and “Escape”. However, for glioblastoma, both tumor endogenous resistance and adaptive resistance play a strong role, including immunosuppressive factor secretion and overexpression of immune checkpoint molecules [6–9], reduced human leukocyte antigen (HLA) levels [10], and increased number of regulatory T cells (Tregs) [11, 12], leading to more difficult immunotherapy compared with other tumors. Currently, less than 10% of neurospoagioma patients respond to immunotherapy [13]. Achieving effective immunotherapy for neurospoagiomas necessitates not only disrupting immune tolerance and eliciting an immune response against tumor antigens, but also surmounting a range of evolving adaptive and acquired immune evasion mechanisms [14]. Immunotherapy targets tumors directly by engaging the immune system, particularly the acquired immune response. Dendritic cells (DCs), recognized as proficient antigen-presenting cells (APCs), hold a significant position in both innate and adaptive immunity [15]. DC vaccines are created using mononuclear cells extracted from the patients’ own bodies. These cells are cultivated in a laboratory setting, loaded with specific tumor antigens, and subsequently administered to the patients through regular injections [16]. Presently, DC vaccines have found extensive application in treating various tumors, including neurospoagioma [17–19]. This review comprehensively outlines the immunomodulatory effects and associated mechanisms of DC vaccines in the context of neurospoagioma. Additionally, it delves into the advancements made in clinical trials, highlighting potential challenges and suggesting novel avenues for the further development of DC vaccines.

## Biology of DCs

**Fig. 1:**
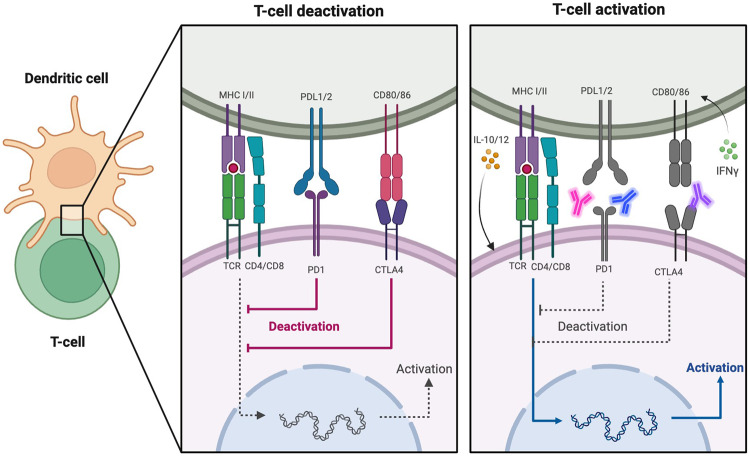
Process of T cell activation with antigen recognition presented by DCs and proper co-stimulation. DCs take up antigens and process them into small peptide segments, which form MHC-antigen complex with MHC, which is presented to the cell surface and binds to T cell receptor (TCR) on the T cell surface as the first signal. Dendritic cells also express the co-stimulatory molecule B7 on the surface, which binds to the corresponding receptor CD28 on the surface of T cells as a second signal; Later, dendritic cells also secrete some cytokines IL10/12 to participate in T cell activation; PDL1/2 binds to PD1 and CD80/86 binds to CTLA4 to inhibit T cell activation.

DCs, initially identified and named by Ralph Steinman in 1973, owe their name to the dendritic or branch-like protrusions that extend from their surface [20]. DC, as a distinctive APC, is found in various anatomical sites and environments, including the dermis and lymph nodes. It possesses the ability to capture, process, and present antigens, as well as activate naive T cells, CD4+ T cells, and B cells, thereby initiating immune responses [21, 22]. DCs can be categorized into mature DCs and immature DCs based on their level of maturation [23]. Immature DCs efficiently capture pathogens, deceased cells, and other antigenic materials from their surroundings, processing them in the process. These DCs exhibit lower expression levels of major histocompatibility complex (MHC)-I, MHC-II, T cell costimulators, and adhesion molecules on their surface. Consequently, their capacity to present antigens and activate T cells is restricted. However, this state can induce immune tolerance by promoting T cell anergy and T cell depletion [24, 25]. Mature DCs exhibit a reduced ability to uptake antigens, yet there is an increased expression of MHC molecules, costimulatory molecules, and adhesion molecules (such as CD40, CD80, CD83, CD54, etc.). Furthermore, these mature DCs secrete and express certain cytokines (such as IL-6, TNF-α, interferon) and chemokine receptors (such as CCR7) [26]. These molecules facilitate the smooth chemotaxis of DCs to peripheral lymphatic organs, promoting interaction with T cells. This interaction allows for the presentation of antigens to T cells, initiating an antigen-specific immune response (Fig. 1).

DC can be categorized into distinct subtypes based on their developmental processes: classical DC (cDC), plasmacytoid DC (pDC), and monocyte-derived DC (moDC) [27]. Conventional dendritic precursor cells (pre-cDC), plasmacytoid DC (pDC), and mononuclear precursor cells are derived from the bone marrow and migrate to various lymphoid and non-lymphoid tissues, including lymph nodes, spleen, skin, lung, and intestine, through the bloodstream. Once in peripheral tissues like the skin, pre-cDC and mononuclear progenitor cells differentiate into immature cDC and moDC, respectively [28]. Additionally, there is a category of innate lymphoid cells (ILCs) that exhibit significant similarities to DCs in terms of their role in innate immune responses and the induction of T-cell responses. ILCs, along with DCs and T cells, form four distinct functional immune modules that intricately interact with each other to initiate and orchestrate T cell responses [29, 30].

The primary role of DCs is to capture and internalize antigens, subsequently presenting these antigens to T lymphocytes following processing. Upon stimulation by DCs, initial CD4+ T cells and CD8+ T cells undergo differentiation, ultimately giving rise to distinct effector T cell populations with varying functions [31, 32]. DCs are also capable of activating and interacting with various cells within the innate immune system, including natural killer (NK) cells, macrophages, and mast cells [33, 34]. Furthermore, DCs play a significant role in modulating humoral immunity through interactions with B lymphocytes or by indirectly promoting the proliferation and differentiation of CD4+ T helper cells [35, 36]. DCs play a pivotal role in initiating immune responses as they activate both cellular and humoral immune systems. This central role positions DCs at the core of antigen presentation and vaccination strategies for cancer treatment [37, 38].

## DC vaccines

At present, two primary techniques are widely employed for DC vaccines preparation. The first involves isolating mononuclear cells from the peripheral blood of patients, initiating culture with the addition of GM-CSF and IL4, and subsequently introducing TNF-α to facilitate maturation. The second method entails obtaining CD34+ precursors mobilized from the patient’s bone marrow through GM-CSF treatment before leukocyte separation. The harvested cells are then expanded in a medium containing GM-CSF, Flt3L, and TNF-α for a duration of 1 week or more to yield mature DCs. Following their infusion into the recipient’s body, these DCs can be loaded with various antigens, including nucleic acids, peptides, proteins, cells, nanoparticles, and more [39]. The difference between DC vaccines applied to different cancers is the difference in the tumor-associated antigen (TAA) used to pulse DC [40].

**Fig. 2:**
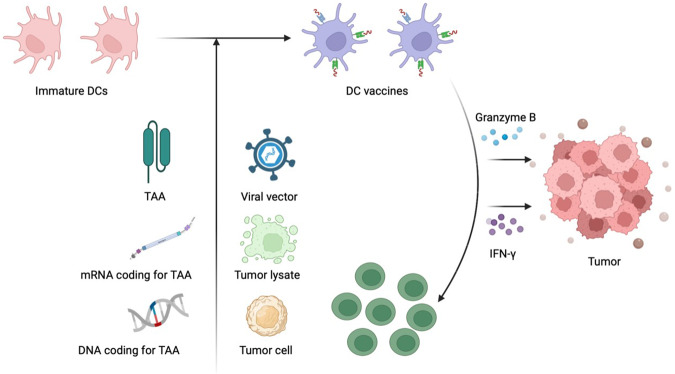
Types of DC-based vaccines and mechanisms to activate antitumor immunity. DC vaccines used for targeted anti-tumor purposes can be broadly categorized into two types: DC polypeptide vaccines and DC gene vaccines. DC polypeptide vaccines encompass: (1) DC vaccines stimulated by tumor antigens; (2) DC vaccines pulsed with tumor cell lysates; (3) DC vaccines created by fusing tumor cells with DCs; (4) DC vaccines loaded with exosomes.

Currently, DC vaccines used for targeted anti-tumor purposes can be broadly categorized into two types: DC polypeptide vaccines and DC gene vaccines. DC polypeptide vaccines encompass: (1) DC vaccines stimulated by tumor antigens; (2) DC vaccines pulsed with tumor cell lysates; (3) DC vaccines created by fusing tumor cells with DCs; (4) DC vaccines loaded with exosomes [41] (Fig. 2). Among these, exosomes loaded with DC vaccines have been demonstrated to exhibit effective anti-tumor immune effects in animal tumor model experiments, thus emerging as a novel focal point in the realm of anti-tumor immunotherapy research [42]. DC gene vaccines involve modifying DC vaccines with tumor DNA, tumor RNA, cytokines, as well as costimulatory and adhesion molecules through transfection.

Tumor immunotherapy is an emerging approach aimed at enhancing the immune system’s function to combat tumors. It has garnered significant attention as an alternative or complementary treatment to traditional methods like surgery, chemotherapy, and radiotherapy [43]. Currently, tumor immunotherapy is broadly categorized into two main approaches: active immunotherapy and passive immunotherapy [44]. DC vaccines are categorized as a form of active immunotherapy, and there are two primary therapeutic approaches. The first involves inducing the differentiation of DCs using various in vitro methods, followed by obtaining autologous T cells with antitumor activity in vitro. These T cells are subsequently reintroduced into patients through intravenous or subcutaneous injection. The second method entails loading DCs with tumor antigens and then administering these modified DCs to the patient. This stimulates the host’s anti-tumor immune response, leading to a specific cytotoxic effect on tumor cells [45].

## DC vaccines in immunomodulation of neurospoagioma

### DC polypeptide vaccine

Sensitizing DCs with tumor antigens and tumor cell lysates represents the most frequently employed technique for generating DC vaccines. This approach involves initially loading immature DCs with tumor antigens obtained from patients. Subsequently, these DCs mature and are re-administered to patients, carrying the antigen information. This process serves to effectively activate the body’s anti-tumor immune response, fostering a targeted immune reaction against the tumor [46]. Cancer stem-like cells (CSCs) have been documented to exhibit elevated levels of TAAs as well as MHC molecules [47]. Hence, researchers have delved into the appropriateness of utilizing CSCs as a reservoir of antigens for DC vaccination against human GBM, with the goal of achieving precise CSC targeting and fortifying anti-tumor immune reactions. In vitro investigations have demonstrated that DC vaccines laden with CSC antigens prompt the production of interferon (IFN)-γ, thereby triggering a CSC-specific T cell response. Subsequent in vivo studies have further substantiated that DC vaccines loaded with CSC antigens can spur the development of cytotoxic T lymphocytes (CTLs) directed at CSCs, resulting in an extended survival period for rats carrying neurospoagioma [48]. These findings underscore the potential of IFN-γ to enhance the immune microenvironment of patients through the initiation of an adaptive immune response. It is worth noting that in 20 distinct human neurospoagioma cell lines, the presence of antigenic peptides such as TRP-2, gp100, human epidermal growth factor receptor 2 (HER-2/neu), and survivin has been identified through flow cytometry analysis, based on their molecular phenotypes and expression of HLA Class I antigens [49]. Prins et al. developed a vaccine consisting of DCs pulsed with neurospoagioma-associated antigen (GAA) peptides and conducted clinical trials. They observed an elevated frequency of NK cells and a reduced rate of Tregs in the peripheral blood of patients who received the GAA-DCs vaccine [50]. This indicates that employing CD25 inhibitors to decrease Treg levels could potentially enhance the effectiveness of the GAA-DCs vaccine. Furthermore, Johanns et al. [51] also also developed a DCs vaccine based on neoantigens using synthetic long peptides. In a patient with GBM, they observed specific CD8+ and CD4+ T cell responses targeting neoantigens following vaccination with the peptide-loaded DCs vaccine. This study utilized an immunogenomics pipeline to identify potential neoantigens and assess neoantigen-specific T cell reactivity, presenting a promising approach for the development of personalized DC vaccines [51]. Continued research is warranted to comprehend the development of resistant subclones after treatment, even in the presence of antigen-specific T cells. Analyzing the neoantigenic and molecular characteristics of the tumor following treatment, as well as investigating the microenvironment, could shed light on potential mechanisms of immune evasion.

Additionally, tumor cell lysates-pulsed DC vaccines have shown promise in the context of neurospoagioma prevention. For instance, Rudnick et al. administered autologous tumor lysates-pulsed DC vaccines to 20 patients with malignant neurospoagioma, resulting in increased IFN-γ responses post-vaccination [52]. This observation suggests that tumor cell lysates-pulsed DC vaccines can also influence the tumor immune response by means of IFN-γ modulation. In a separate investigation, Benitez-Ribas et al. developed a vaccine using neurospoagioma cell line lysates that were pulsed onto autologous DCs. Subsequently, these tumor cell lysates-pulsed DC vaccines were subjected to in vitro co-culturing with peripheral blood mononuclear cells (PBMCs), revealing an enhanced specific T cell response [53]. Interestingly, Parney et al. [54]. introduced an innovative approach for creating autologous tumor lysate-pulsed DC vaccines targeting GBM. In this technique, a platelet lysate-based supplement was utilized to establish human GBM cell lines. GBM cell lines established with the platelet lysate supplement exhibited accelerated growth and higher expression of neurospoagioma-associated antigens compared to lines cultivated in neural stem cell media or serum-containing media. Unlike conventional culture methods, the refined technique developed by Parney et al. yielded substantial quantities of mature CD83+ DCs derived from monocytes of GBM patients. Autologous T cells, stimulated with mature DCs loaded with allogeneic GBM cell line lysate, displayed robust cytotoxic activity against HLA-A2-matched GBM cells [54]. Furthermore, Bacterial Ghosts have been identified to retain intact cell surface structures, displaying potent adjuvant properties crucial for inducing DC maturation. These Ghosts possess an empty internal compartment that can be readily loaded with tumor antigens, such as tumor lysate, facilitating the optimal generation of therapeutic DC vaccines [55]. Significantly, a recent study has highlighted the activation of miR-106a/20b as a potentially crucial molecular mechanism that enhances antitumor immune responses in neurospoagioma stem cell-mediated DCs. This activation leads to the downregulation of STAT3 expression, thereby alleviating its inhibitory effect [56]. These findings suggest a potential involvement of miRNA and STAT3 signaling pathways in regulating the activation of immune responses against tumor cell lysates-pulsed DC vaccines. The clinical significance lies in the personalized nature of these vaccines, targeting a spectrum of tumor antigens tailored to individual cancer profiles. Moreover, the utilization of Bacterial Ghosts as adjuvants adds a unique dimension to enhance vaccine immunogenicity. Looking forward, optimizing vaccine formulations, expanding large-scale clinical trials, unraveling molecular mechanisms, and exploring combination therapies are key directions for future research. Technological advances, including single-cell omics and advanced imaging, offer avenues for a deeper understanding of immune responses post-vaccination. The ultimate goal is to harness the full potential of tumor cell lysates-pulsed DC vaccines, offering a targeted and effective immunotherapeutic strategy for diverse cancer types.

### DC gene vaccines

We conducted further exploration into the immune regulation of neurospoagioma through DC gene vaccines. CD133, a cell surface antigen, has been identified on the cancer stem cells of various solid tumors, including neurospoagioma [57, 58]. The enrichment of CD133-positive neurospoagioma stem cells has been linked to a highly tumorigenic population [59, 60], which shows a negative correlation with patient survival. This characteristic makes these cells an ideal target for focused immunotherapy [61]. In a study by Do et al., a groundbreaking humanized mouse model was established, demonstrating the robust and simultaneous activation of CD8+ and CD4+ T cells through CD133 mRNA-loaded DC vaccination. This resulted in a potent and durable immune response, subsequently leading to the inhibition of CD133-positive neurospoagioma stem cell proliferation and suppression of tumor growth [62]. In another study, tumor samples obtained from patients with GBM were initially examined to identify TAA that were overexpressed. Subsequently, DC gene vaccines were formulated by transfecting autologous dendritic cells with pre-fabricated messenger RNA (mRNA) encoding the complete sequence of the identified TAA [63]. The developed DC vaccines were observed to elicit specific immune responses from CD4+ and/or CD8+ T cells targeting the antigen [63]. These findings provide support for the capacity of DC gene vaccines to trigger an anti-tumor immune response in neurospoagioma. Moreover, Sayour et al. devised clinically applicable nanoliposomes capable of binding to tumor-derived RNAs, generating individualized tumor RNA-nanoparticles (NPs) with considerable scalability. These RNA-NPs circumvent MHC limitations, aid in central distribution, and offer rapid immune activation [64]. Subsequently, RNA-NPs were demonstrated to elevate the expression percentage of MHC class I/II, B7 co-stimulatory molecules, and maturation markers on DCs, leading to a robust expansion of antigen-specific T cells [64]. Targeting DCs with nanoparticles or exosomes for RNA delivery holds great promise as a strategy for developing DC gene vaccines. This has implications for improving the feasibility and effectiveness of DC gene vaccines by addressing challenges associated with antigen presentation and immune response amplification. The clinical value lies in the potential for developing a broadly applicable and patient-specific immunotherapeutic strategy for neurospoagioma. Future directions in research could focus on optimizing and standardizing these approaches for broader clinical implementation. Investigating the broader applicability of CD133 as a target, exploring additional tumor-associated antigens, and refining the engineering of DC gene vaccines can contribute to enhancing their efficacy and minimizing potential side effects. Additionally, the translation of these approaches from preclinical models to clinical trials is a critical step in establishing their safety and effectiveness in neurospoagioma patients.

## Clinical application of DC vaccines in neurospoagioma

DC vaccines have demonstrated their potential in modulating the immune response against neurospoagioma. Nevertheless, their clinical applicability and safety necessitate further investigation. In the context of tumor antigens-stimulated DC vaccines, Iwami et al. conducted a phase I clinical trial involving DC vaccination in patients with recurrent malignant neurospoagiomas. The trial utilized two tumor-derived peptides, which were restricted to HLA-A0201 and -A2402, respectively [65]. The findings suggest the feasibility and safety of the regimen, and the HLA-A*24-restricted peptide has shown the capability to elicit an immune response. These results warrant further investigation in a subsequent study to assess whether the inclusion of postoperative chemotherapy can effectively delay relapse in patients diagnosed with newly malignant neurospoagiomas [65].

Regarding tumor cell lysates-pulsed DC vaccines, a preliminary phase I/II clinical study involved 18 patients diagnosed with grade 4 GBM who underwent DC vaccination with an autologous tumor lysate pulse. Encouragingly, the vaccinated group exhibited notably improved survival rates compared to the 27 non-vaccinated controls. Furthermore, no severe adverse effects were observed among any of the patients, and there was no clinical or radiological evidence of autoimmune reactions [66]. This study provided valuable insights into the safety and clinical efficacy of autologous tumor lysate-pulsed DC vaccines in the context of malignant neurospoagioma. Another noteworthy clinical investigation, a multicohort dose-escalation study [67], involved the treatment of twelve GBM patients with varying doses of autologous DCs pulsed with autologous tumor peptide, specifically 1, 5, or 10 million cells. The study observed measurable systemic antitumor CTL responses. Notably, the administration of DC vaccination did not result in any evidence of dose-limiting toxicity or severe adverse reactions [67]. In a recent Phase 3 prospective externally controlled non-randomized trial, the overall survival rate (OS) of GBM patients who received treatment with tumor lysate-loaded DC vaccine (DCVax-L) in addition to the standard of care (SOC) was compared to that of externally matched patients who underwent SOC alone over the same period [68]. The study revealed that the inclusion of DCVax-L along with SOC led to a significant and clinically meaningful extension of survival among GBM patients when compared to contemporaneous, matched external controls who solely received SOC (NCT00045968) [68]. Furthermore, the effectiveness and safety of combining tumor cell lysates-pulsed DC vaccines with chemoradiotherapy in treating neurospoagioma were examined. Fadul et al. [69] performed DC vaccination on a cohort of 10 patients diagnosed with GBM who had previously undergone radiotherapy and temozolomide (TMZ) chemotherapy. The study’s findings demonstrated that the combination of DC vaccination with radiotherapy and chemotherapy appears to be both viable and safe for GBM patients, and may also trigger a tumor-specific immune response. Additionally, results from an alternate phase I trial focusing on recurrent GBM highlighted the safety and tolerability of administering temozolomide (TMZ) in conjunction with tumor cell lysates-pulsed DC vaccines [70]. Subsequently, a phase 2 trial was undertaken, targeting individuals who had undergone substantial or near-total tumor resection. The schedule for DC vaccination commenced prior to radiotherapy and persisted throughout adjuvant chemotherapy [71]. The outcomes of this study indicate that the integration of autologous tumor lysate-pulsed DC vaccination with tumor resection and combined radio-chemotherapy is both attainable and secure (NCT01006044). This approach encompasses a distinct vaccine sequence compared to the previous study, thereby facilitating a more comprehensive analysis of the vaccine’s mechanism of action. The feasibility of a multicenter randomized clinical trial is underscored to assess the potential survival advantage conferred by this therapeutic strategy. It is noteworthy that Prins et al. conducted a comparative analysis of the effectiveness and safety of DC vaccines loaded with GAA peptides versus those loaded with autologous tumor lysates (ATL) in patients with GBM [50]. The findings indicate that both modes of DC vaccination carrying different tumor antigens are well-tolerated by patients with glioblastoma, demonstrating no occurrence of dose-limiting toxicity. Notably, the DC vaccination involving ATL pulsing seems to induce a more varied and heterogeneous antitumor immune response against GBM (NCT00068510, NCT00612001) [50].

In the realm of DC gene vaccines, Batich and colleagues devised a unique approach by designing a DC vaccine loaded with Cytomegalovirus (CMV) pp65 RNA. They subsequently implemented three distinct CMV-specific DC vaccine interventions in patients newly diagnosed with GBM. Importantly, each successive study featured a nearly doubled sample size, allowing for a more comprehensive exploration of the vaccine’s potential effects [72]. Subsequent data from the initial blinded, randomized phase II clinical trial (NCT00639639) disclosed that approximately one-third of the participants remain free from tumor recurrence at the five-year mark following diagnosis. In a separate clinical trial (NCT00639639), a 36% survival rate was achieved at the five-year interval from diagnosis. The outcomes of the initial two-arm trial (NCT00639639) revealed an augmented migration of the DC vaccine towards the draining lymph nodes, and this heightened migration phenomenon has been replicated in our more extensive corroborative clinical investigation (NCT02366728) [72]. The consistency of these findings across three successive clinical trials and the extended monitoring of treated patients provides substantial evidence that CMV-specific DC gene vaccines confer a durable survival advantage in the immunotherapy of GBM (Table 1).

**Table 1:** Clinical application of DC vaccines in neurospoagioma.

Registration number	Study Phase	Study design	Data of study start	Sample size	Primary end point	Type pf DC vaccine	Progress	Ref.
/	I	Non-randomized	2007.01	8	Clinical response/toxicity	Tumor antigens-stimulated DC vaccines	Finished	[65]
/	I/II	Non-randomized	/	18	ORR/OS/safety	Tumor cell lysates-pulsed DC vaccines	Finished	[66]
/	I	Non-randomized	/	12	Adverse events/survival	Tumor cell lysates-pulsed DC vaccines	Finished	[67]
NCT00045968	III	RCT	2006.12	331	OS	Tumor cell lysates-pulsed DC vaccines	Ongoing	[68]
ACTRN12611000029998	I	Non-randomized	2009.02	14	PFS/safety	Tumor cell lysates-pulsed DC vaccines	Finished	[70]
NCT01006044	II	Non-randomized	2009.10	32	OS/PFS	Tumor cell lysates-pulsed DC vaccines	Finished	[71]
NCT00068510/NCT00612001	II	Non-randomized	2003.06	34	Adverse events/OS	DC polypeptide vaccine	Finished	[50]
NCT00639639	II	RCT	2002.02	42	OS/safety	DC gene vaccines	Finished	[72]
NCT02366728	II	RCT	2015.10	64	OS	DC gene vaccines	Finished	[72]

## Conclusions and future challenges

DCs stand as the most proficient APCs within the body, demonstrating remarkable efficiency in capturing, processing, and presenting antigens. Notably, they are the sole known APCs capable of directly initiating activation of naïve T cells, thereby fostering a sustained and tumor-specific immune response. By supplying functional DCs to individuals with neurospoagioma, DC vaccines emerge as a potentially safe and effective avenue for immunotherapy against this condition. This review underscores the potential of DC vaccines to modulate the tumor microenvironment in neurospoagioma, with numerous clinical trials affirming their capacity to induce targeted immune responses, all while maintaining a favorable toxicity profile. However, it is pertinent to note that despite these promising outcomes, DC vaccine products for neurospoagioma therapy are not yet commercially available. A few challenges persist on the path to realizing DC vaccine products for neurospoagioma treatment. The translation of these findings into practical clinical applications demands further research and development efforts, as well as rigorous validation in larger-scale clinical trials. Nonetheless, the substantial progress achieved thus far in harnessing the power of DC vaccines underscores their potential as a valuable tool in the immunotherapy arsenal against neurospoagioma.

Currently, the preparation of DC vaccines offers flexibility in choosing various approaches, including selecting individual epitopes, combining multiple epitopes, or utilizing total tumor mRNA or tumor cell lysate for loading onto DCs. The careful selection of appropriate vaccine targets holds immense significance for disease prevention and treatment. However, in certain cases like some tumors or viral infections such as HCV, identifying clear-cut targets remains a challenge. Striking the right balance is crucial; an excessive number of targets might lead to autoimmune reactions, while too few could result in limited or ineffective outcomes. To address this issue, extensive efforts are underway, such as the exploration of neoantigens, to surmount these challenges. The discovery and implementation of effective and well-defined disease targets for DC vaccines could significantly expedite their development. As research progresses and novel targets are identified, the potential for rapid advancement in the field of DC vaccine technology becomes increasingly promising. This underscores the ongoing commitment to refining and enhancing the efficacy of DC vaccines in disease management.

Another crucial aspect to consider is the diverse range of outcomes resulting from various delivery methods employed during the in vitro induction of DCs. Notably, distinct modes of administration, including intradermal, subcutaneous, and venous delivery, can yield differing consequences. For instance, with intradermal and subcutaneous delivery approaches, DCs exhibit a tendency to migrate towards the draining lymph nodes, albeit with a relatively limited mobility, typically around 5% [73]. Conversely, when employing the vein delivery mode, DCs tend to accumulate within tissues and organs such as the liver, spleen, and kidney, albeit with relatively modest effectiveness [74]. Therefore, the selection and optimization of the delivery system is a problem to be solved in the future.

Furthermore, a significant hurdle in the widespread adoption of DC vaccines pertains to the scarcity of DCs within the human body and the inherent immunological barriers that exist between individuals. These challenges necessitate a personalized and individualized approach to the preparation of DC vaccines, giving rise to a host of complexities that impede their broader applicability. Moreover, the efficient expansion and cultivation of autologous DCs, ensuring their uniformity and adherence to standardized protocols in the production of DC vaccines, and the establishment of robust evaluation criteria for assessing their effectiveness are all pressing concerns that must be addressed to facilitate the industrialization and large-scale implementation of DC vaccines. Tackling these issues head-on is imperative to surmount the obstacles inherent in the production, distribution, and utilization of DC vaccines, thereby paving the way for their successful integration into mainstream medical practice. Only by achieving these milestones can the full potential of DC-based immunotherapies be realized in the context of neurospoagioma treatment and beyond.

The DC vaccine represents a novel and promising approach to vaccination that has garnered significant interest in recent years. Operating from the vantage point of antigen presentation, this innovative vaccine holds substantial potential for both disease prevention and treatment. Moving forward, it remains crucial to delve further into the underlying foundational principles, delving into these theories with greater depth and meticulous detail. Continued research efforts are essential for a comprehensive understanding of the intricate immune regulatory network involving DCs. This exploration should extend to the broader array of cells participating in immune processes, thereby bolstering the advancement of DC vaccine development within the realm of neurospoagioma therapy. By charting these scientific frontiers, we can catalyze the refinement and optimization of DC vaccines, ultimately enhancing their efficacy and impact on combating neurospoagioma and other diseases.

## References

[1] Yan Y, Dai W, Mei Q (2022). Multicentric glioma: an ideal model to reveal the mechanism of glioma. Front Oncol.

[2] Ostrom QT, Cioffi G, Waite K, Kruchko C, Barnholtz-Sloan JS (2021). CBTRUS statistical report: primary brain and other central nervous system tumors diagnosed in the United States in 2014-2018. Neuro Oncol.

[3] van Solinge TS, Nieland L, Chiocca EA, Broekman MLD (2022). Advances in local therapy for glioblastoma—taking the fight to the tumour. Nat Rev Neurol.

[4] Banchereau J, Schuler-Thurner B, Palucka AK, Schuler G (2001). Dendritic cells as vectors for therapy. Cell.

[5] Dunn GP, Old LJ, Schreiber RD (2004). The three Es of cancer immunoediting. Annu Rev Immunol.

[6] Nitta T, Hishii M, Sato K, Okumura K (1994). Selective expression of interleukin-10 gene within glioblastoma multiforme. Brain Res.

[7] Sawamura Y, Diserens AC, de Tribolet N (1990). In vitro prostaglandin E2 production by glioblastoma cells and its effect on interleukin-2 activation of oncolytic lymphocytes. J Neurooncol.

[8] Couldwell WT, Yong VW, Dore-Duffy P, Freedman MS, Antel JP (1992). Production of soluble autocrine inhibitory factors by human glioma cell lines. J Neurol Sci.

[9] Rorive S, Belot N, Decaestecker C, Lefranc F, Gordower L, Micik S (2001). Galectin-1 is highly expressed in human gliomas with relevance for modulation of invasion of tumor astrocytes into the brain parenchyma. Glia.

[10] Facoetti A, Nano R, Zelini P, Morbini P, Benericetti E, Ceroni M (2005). Human leukocyte antigen and antigen processing machinery component defects in astrocytic tumors. Clin Cancer Res.

[11] Fecci PE, Mitchell DA, Whitesides JF, Xie W, Friedman AH, Archer GE (2006). Increased regulatory T-cell fraction amidst a diminished CD4 compartment explains cellular immune defects in patients with malignant glioma. Cancer Res.

[12] Jacobs JF, Idema AJ, Bol KF, Nierkens S, Grauer OM, Wesseling P (2009). Regulatory T cells and the PD-L1/PD-1 pathway mediate immune suppression in malignant human brain tumors. Neuro Oncol.

[13] Huang X, Shi S, Wang H, Zhao T, Wang Y, Huang S (2023). Advances in antibody-based drugs and their delivery through the blood-brain barrier for targeted therapy and immunotherapy of gliomas. Int Immunopharmacol.

[14] Babington K (1987). Sense and sensitivity. N Z Nurs J.

[15] Niedbala M, Malarz K, Sharma G, Kramer-Marek G, Kaspera W (2022). Glioblastoma: pitfalls and opportunities of immunotherapeutic combinations. Onco Targets Ther.

[16] Fu C, Ma T, Zhou L, Mi QS, Jiang A (2022). Dendritic cell-based vaccines against cancer: challenges, advances and future opportunities. Immunol Invest.

[17] Huang L, Liu Z, Wu C, Lin J, Liu N (2023). Magnetic nanoparticles enhance the cellular immune response of dendritic cell tumor vaccines by realizing the cytoplasmic delivery of tumor antigens. Bioeng Transl Med.

[18] Lepski G, Bergami-Santos PC, Pinho MP, Chauca-Torres NE, Evangelista GCM, Teixeira SF (2023). Adjuvant vaccination with allogenic dendritic cells significantly prolongs overall survival in high-grade gliomas: results of a phase II trial. Cancers (Basel).

[19] Dwivedi R, Pandey R, Chandra S, Mehrotra D (2023). Dendritic cell-based immunotherapy: a potential player in oral cancer therapeutics. Immunotherapy.

[20] Rowley DA, Fitch FW (2012). The road to the discovery of dendritic cells, a tribute to Ralph Steinman. Cell Immunol.

[21] Balan S, Saxena M, Bhardwaj N (2019). Dendritic cell subsets and locations. Int Rev Cell Mol Biol.

[22] Wang Y, Xiang Y, Xin VW, Wang XW, Peng XC, Liu XQ (2020). Dendritic cell biology and its role in tumor immunotherapy. J Hematol Oncol.

[23] Cella M, Sallusto F, Lanzavecchia A (1997). Origin, maturation and antigen presenting function of dendritic cells. Curr Opin Immunol.

[24] Dalod M, Chelbi R, Malissen B, Lawrence T (2014). Dendritic cell maturation: functional specialization through signaling specificity and transcriptional programming. EMBO J.

[25] Anguille S, Smits EL, Lion E, van Tendeloo VF, Berneman ZN (2014). Clinical use of dendritic cells for cancer therapy. Lancet Oncol.

[26] Benteyn D, Heirman C, Bonehill A, Thielemans K, Breckpot K (2015). mRNA-based dendritic cell vaccines. Expert Rev Vaccines.

[27] Wculek SK, Cueto FJ, Mujal AM, Melero I, Krummel MF, Sancho D (2020). Dendritic cells in cancer immunology and immunotherapy. Nat Rev Immunol.

[28] Liu J, Zhang X, Cheng Y, Cao X (2021). Dendritic cell migration in inflammation and immunity. Cell Mol Immunol.

[29] Bagadia P, Huang X, Liu TT, Murphy KM (2019). Shared transcriptional control of innate lymphoid cell and dendritic cell development. Annu Rev Cell Dev Biol.

[30] Briseno CG, Murphy TL, Murphy KM (2014). Complementary diversification of dendritic cells and innate lymphoid cells. Curr Opin Immunol.

[31] Banchereau J, Steinman RM (1998). Dendritic cells and the control of immunity. Nature.

[32] Hilligan KL, Ronchese F (2020). Antigen presentation by dendritic cells and their instruction of CD4+ T helper cell responses. Cell Mol Immunol.

[33] Steinman RM (2012). Decisions about dendritic cells: past, present, and future. Annu Rev Immunol.

[34] Steinman RM, Banchereau J (2007). Taking dendritic cells into medicine. Nature.

[35] Jego G, Pascual V, Palucka AK, Banchereau J (2005). Dendritic cells control B cell growth and differentiation. Curr Dir Autoimmun.

[36] Qi H, Egen JG, Huang AY, Germain RN (2006). Extrafollicular activation of lymph node B cells by antigen-bearing dendritic cells. Science.

[37] Hwang H, Shin C, Park J, Kang E, Choi B, Han JA (2016). Human breast cancer-derived soluble factors facilitate CCL19-induced chemotaxis of human dendritic cells. Sci Rep.

[38] Bai X, Zhou Y, Yokota Y, Matsumoto Y, Zhai B, Maarouf N (2022). Adaptive antitumor immune response stimulated by bio-nanoparticle based vaccine and checkpoint blockade. J Exp Clin Cancer Res.

[39] Qian D, Li J, Huang M, Cui Q, Liu X, Sun K (2023). Dendritic cell vaccines in breast cancer: Immune modulation and immunotherapy. Biomed Pharmacother.

[40] Mody N, Dubey S, Sharma R, Agrawal U, Vyas SP (2015). Dendritic cell-based vaccine research against cancer. Expert Rev Clin Immunol.

[41] Yu J, Sun H, Cao W, Song Y, Jiang Z (2022). Research progress on dendritic cell vaccines in cancer immunotherapy. Exp Hematol Oncol.

[42] Ren WN, Chang CK, Fan HH, Guo F, Ren YN, Yang J (2011). A combination of exosomes carrying TSA derived from HLA-A2-positive human white buffy coat and polyI:C for use as a subcellular antitumor vaccination. J Immunoassay Immunochem.

[43] Palucka K, Banchereau J (2012). Cancer immunotherapy via dendritic cells. Nat Rev Cancer.

[44] Liu S, Sun Q, Ren X (2023). Novel strategies for cancer immunotherapy: counter-immunoediting therapy. J Hematol Oncol.

[45] Lu Y, You J (2023). Strategy and application of manipulating DCs chemotaxis in disease treatment and vaccine design. Biomed Pharmacother.

[46] Oosterwijk-Wakka JC, Tiemessen DM, Bleumer I, de Vries IJ, Jongmans W, Adema GJ (2002). Vaccination of patients with metastatic renal cell carcinoma with autologous dendritic cells pulsed with autologous tumor antigens in combination with interleukin-2: a phase 1 study. J Immunother.

[47] Pellegatta S, Poliani PL, Corno D, Menghi F, Ghielmetti F, Suarez-Merino B (2006). Neurospheres enriched in cancer stem-like cells are highly effective in eliciting a dendritic cell-mediated immune response against malignant gliomas. Cancer Res.

[48] Xu Q, Liu G, Yuan X, Xu M, Wang H, Ji J (2009). Antigen-specific T-cell response from dendritic cell vaccination using cancer stem-like cell-associated antigens. Stem Cells.

[49] Zhang JG, Eguchi J, Kruse CA, Gomez GG, Fakhrai H, Schroter S (2007). Antigenic profiling of glioma cells to generate allogeneic vaccines or dendritic cell-based therapeutics. Clin Cancer Res.

[50] Prins RM, Wang X, Soto H, Young E, Lisiero DN, Fong B (2013). Comparison of glioma-associated antigen peptide-loaded versus autologous tumor lysate-loaded dendritic cell vaccination in malignant glioma patients. J Immunother.

[51] Johanns TM, Miller CA, Liu CJ, Perrin RJ, Bender D, Kobayashi DK (2019). Detection of neoantigen-specific T cells following a personalized vaccine in a patient with glioblastoma. Oncoimmunology.

[52] Rudnick JD, Sarmiento JM, Uy B, Nuno M, Wheeler CJ, Mazer MJ (2020). A phase I trial of surgical resection with Gliadel Wafer placement followed by vaccination with dendritic cells pulsed with tumor lysate for patients with malignant glioma. J Clin Neurosci.

[53] Benitez-Ribas D, Cabezon R, Florez-Grau G, Molero MC, Puerta P, Guillen A (2018). Immune response generated with the administration of autologous dendritic cells pulsed with an allogenic tumoral cell-lines lysate in patients with newly diagnosed diffuse intrinsic pontine glioma. Front Oncol.

[54] Parney IF, Gustafson MP, Solseth M, Bulur P, Peterson TE, Smadbeck JB (2020). Novel strategy for manufacturing autologous dendritic cell/allogeneic tumor lysate vaccines for glioblastoma. Neurooncol Adv.

[55] Dobrovolskiene N, Pasukoniene V, Darinskas A, Krasko JA, Zilionyte K, Mlynska A (2018). Tumor lysate-loaded bacterial ghosts as a tool for optimized production of therapeutic dendritic cell-based cancer vaccines. Vaccine.

[56] Zhou H, Sun C, Li C, Hua S, Li F, Li R (2022). The microRNA-106a/20b strongly enhances the antitumour immune responses of dendritic cells pulsed with glioma stem cells by targeting STAT3. J Immunol Res.

[57] Petsri K, Yokoya M, Racha S, Thongsom S, Thepthanee C, Innets B (2023). Novel synthetic derivative of renieramycin T right-half analog induces apoptosis and inhibits cancer stem cells via targeting the Akt signal in lung cancer cells. Int J Mol Sci.

[58] Zhou T, Man Q, Li X, Xie Y, Hou X, Wang H (2023). Artificial intelligence-based comprehensive analysis of immune-stemness-tumor budding profile to predict survival of patients with pancreatic adenocarcinoma. Cancer Biol Med.

[59] Gallo M, Ho J, Coutinho FJ, Vanner R, Lee L, Head R (2013). A tumorigenic MLL-homeobox network in human glioblastoma stem cells. Cancer Res.

[60] Li Z, Bao S, Wu Q, Wang H, Eyler C, Sathornsumetee S (2009). Hypoxia-inducible factors regulate tumorigenic capacity of glioma stem cells. Cancer Cell.

[61] Kase M, Minajeva A, Niinepuu K, Kase S, Vardja M, Asser T (2013). Impact of CD133 positive stem cell proportion on survival in patients with glioblastoma multiforme. Radiol Oncol.

[62] Do ASS, Amano T, Edwards LA, Zhang L, De Peralta-Venturina M, Yu JS (2020). CD133 mRNA-loaded dendritic cell vaccination abrogates glioma stem cell propagation in humanized glioblastoma mouse model. Mol Ther Oncolytics.

[63] Wang QT, Nie Y, Sun SN, Lin T, Han RJ, Jiang J (2020). Tumor-associated antigen-based personalized dendritic cell vaccine in solid tumor patients. Cancer Immunol Immunother.

[64] Sayour EJ, De Leon G, Pham C, Grippin A, Kemeny H, Chua J (2017). Systemic activation of antigen-presenting cells via RNA-loaded nanoparticles. Oncoimmunology.

[65] Iwami K, Shimato S, Ohno M, Okada H, Nakahara N, Sato Y (2012). Peptide-pulsed dendritic cell vaccination targeting interleukin-13 receptor alpha2 chain in recurrent malignant glioma patients with HLA-A*24/A*02 allele. Cytotherapy.

[66] Yamanaka R, Homma J, Yajima N, Tsuchiya N, Sano M, Kobayashi T (2005). Clinical evaluation of dendritic cell vaccination for patients with recurrent glioma: results of a clinical phase I/II trial. Clin Cancer Res.

[67] Liau LM, Prins RM, Kiertscher SM, Odesa SK, Kremen TJ, Giovannone AJ (2005). Dendritic cell vaccination in glioblastoma patients induces systemic and intracranial T-cell responses modulated by the local central nervous system tumor microenvironment. Clin Cancer Res.

[68] Liau LM, Ashkan K, Brem S, Campian JL, Trusheim JE, Iwamoto FM (2023). Association of autologous tumor lysate-loaded dendritic cell vaccination with extension of survival among patients with newly diagnosed and recurrent glioblastoma: a phase 3 prospective externally controlled cohort trial. JAMA Oncol.

[69] Fadul CE, Fisher JL, Hampton TH, Lallana EC, Li Z, Gui J (2011). Immune response in patients with newly diagnosed glioblastoma multiforme treated with intranodal autologous tumor lysate-dendritic cell vaccination after radiation chemotherapy. J Immunother.

[70] Hunn MK, Bauer E, Wood CE, Gasser O, Dzhelali M, Ancelet LR (2015). Dendritic cell vaccination combined with temozolomide retreatment: results of a phase I trial in patients with recurrent glioblastoma multiforme. J Neurooncol.

[71] Inoges S, Tejada S, de Cerio AL, Gallego Perez-Larraya J, Espinos J, Idoate MA (2017). A phase II trial of autologous dendritic cell vaccination and radiochemotherapy following fluorescence-guided surgery in newly diagnosed glioblastoma patients. J Transl Med.

[72] Batich KA, Mitchell DA, Healy P, Herndon JE, Sampson JH (2020). Once, twice, three times a finding: reproducibility of dendritic cell vaccine trials targeting cytomegalovirus in glioblastoma. Clin Cancer Res.

[73] Swartz AM, Hotchkiss KM, Nair SK, Sampson JH, Batich KA (2022). Generation of tumor targeted dendritic cell vaccines with improved immunogenic and migratory phenotype. Methods Mol Biol.

[74] Gardner A, de Mingo Pulido A, Ruffell B (2020). Dendritic cells and their role in immunotherapy. Front Immunol.

